# Ceria Boosting on *In Situ* Nitrogen-Doped Graphene Oxide for Efficient Bifunctional ORR/OER Activity

**DOI:** 10.3389/fchem.2022.889579

**Published:** 2022-06-24

**Authors:** L. Kashinath, K. Byrappa

**Affiliations:** ^1^ Centre for Materials Science and Technology, University of Mysore, Mysore, India; ^2^ Experimental Physics Lab, Division of Materials Science, Department of Engineering Science and Mathematics, Lulea University of Technology, Lulea, Sweden; ^3^ Adichunchanagiri University, Mandya, India

**Keywords:** OER, ORR (oxygen reduction reaction), electrocatalyst, heterostructures, microwave

## Abstract

In the present work, a highly efficient and excellent electrocatalyst material for bifunctional oxygen reduction/evolution reaction (ORR/OER) was synthesized using the microwave-assisted hydrothermal method. In brief, ultrafine hexagonal cerium oxide (CeO_2_) nanoparticles were tailored on the layered surface of *in situ* nitrogen-doped graphene oxide (NGO) sheets. The nanocomposites exhibited a high anodic onset potential of 0.925 V vs. RHE for ORR activity and 1.2 V for OER activity with a very high current density in 0.5 M KOH. The influence of oxygen cluster on Ce^3+^/Ce^4+^ ion decoration on outward/inward *in situ* nitrogen-coupled GO enhanced the physicochemical properties of composites and in turn increased electron transferability. The microwave-assisted hydrothermal coupling technique provides a higher density, active sites on CeO_2_@NGO composites, and oxygen deficiency structures in ultrafine Ce-O particles and boosts higher charge transferability in the composites. It is believed that the physical states of Ce-N- C, Ce-C=O, and a higher amount of oxygen participation with ceria increase the density of composites that in turn increases the efficiency. N-doped graphene oxide promotes high current conduction and rapid electron transferability while reducing the external transport resistance in oxygen electrocatalysis by sufficient mass transfer through in-built channels. This study may provide insights into the knowledge of Ce-enabled bifunctional activity to guide the design of a robust catalyst for electrochemical performance.

## 1 Introduction

Fuel cells and efficient renewable energy technologies for storage are the promising energy conversion methods for meeting the energy demands of today’s world, owing to their low environmental impact; high stability and specific density are the growing ecological concerns (Turner, 2004). The increasing demands of energy and environmental concern are a focused interest for recreating fuel cells with high energy and high power density with utmost performance using low-cost nonprecious materials that are highly needed in current research trends (Spendelow and Wieckowski, 2007; McKone et al., 2011). The fabrication of low-cost and non-precious bifunctional electrocatalysts for OER/ORR is a vital component for enhancing the energy-related and environmental issues for the replacement of precious metals.(Yeager, 1984; Spendelow and Wieckowski, 2007). An important technical issue is to get rid of the high polarization of cathodic materials during charging (ORR) and discharging activities (OER) (Li et al., 2013), (Yang et al., 2015), (Fan et al., 2015). The oxygen reduction/evolution reaction is the significant feedback for energy (fuel cells) application processes which requires a clean and sustainable technique for the development of active materials for excellent energy-related works (Zhu and Dong, 2013; Sun et al., 2015). In recent studies, the physicochemical properties of the two-dimension framework of nitrogenized graphene oxide showed excellent tuning of physical and chemical properties of the composites for many potential applications. The nitrogen-containing graphene metal oxide nanocomposites are extensively explored for different potential applications due to enhanced physical/chemical characteristics and in turn can be tuned or altered easily for many potential applications with fine structure and morphology.

Recent studies have proved that the incorporation of metal oxides (transition) into nitrogen-coupled graphene nanosheets have been replaced as the promising materials for the precious noble metal catalyst for electrochemical studies acquiring unique features of charge transfer and metallic interface and having high synergetic effects between the N-C and metal atoms (Tsai et al., 2015). In addition, the combination of nitrogen and GO can increase the active absorbing sites on the surface which can supportively interact with the metal ions and in turn, increase the redox reaction due to the physiochemical bonding of NGO and the metal ion nanoparticles which plays a vital role in triggering the Faradic reaction, and in other sense, nitrogen-doped carbon bonding may lead to carbon nitride clusters that promote high electrical conductivity (Zhou et al., 2000; Ling et al., 2013; Xu et al., 2020). This coupled NGO showed a better synergetic effect and increased the lifespan without losing its stability and high retention capability which attracted the researchers (Zhang et al., 2009; Guo et al., 2012; Saravanan et al., 2015). Among oxides, CeO_2_ is an anodic material for LIB, electrochemical, energy, and environmental applications and has rapid transformation activity between Ce (IV) and Ce (III). Cerium oxide interaction with graphene-based materials with large-scale *ab initio* calculations is seen (Zafiris and Gorte, 1993; Dowding et al., 2012; Zhang et al., 2015). Cerium oxide can be readily oxidized and reduced due to high oxygen content, and redox properties can be initiated by a metal surface leak over from ceria (Nolan, 2012; Saravanan et al., 2015) and have utilization in ethanol/ethanol-water conversion into hydrogen. Many reports showed that a greater charge transfer ratio of GO to ceria is a work function difference and proportional to the sum of charge (transferred). Additionally, the concentration of oxygen atoms on GO sheets makes strong interaction between CeO_2_ and GO sheets (Kumar S. et al., 2016). The nitrogen coordination with the graphene-based catalyst for OER/ORR with ceria nanoparticles is not yet reported. This frame of the network has a nitrogen-rich content that promotes the electron transfer and conduction, increment in the surface extension, and porosity that generates higher power with long-run stability in the acidic and alkaline media.

In the present work, we developed highly crystalline hexagonal CeO_2_ nanoparticles on the layered surface of NGO by microwave-assisted hydrothermal processing and reported as the newest and successfully tested technique for bifunctional ORR/OER activities. The Ce^3+^/Ce^4+^ ion decoration on the outside of *in situ* nitrogen-coupled GO was successfully utilized as the electrocatalyst material for ORR/OER. The embedding of CeO2 nanoparticles in deep on both sides of the layered surface of graphene sheets prevents the nanoparticles from accumulation, refining charge tendency, and electron conductivity (Wang et al., 2011; Kalubarme et al., 2012). The rich phase of nitrogen on graphene oxide effectively incorporates the controlled nanoparticles of ceria and facilitates the inactive sites, and a higher amount of oxygen participation with ceria transition leads to higher power production in rapid transfer, and enhanced physicochemical properties are highly motivated bifunctional oxygen reaction activity (Hummers and Offeman, 1958; Peng et al., 2016). Thus, the combination of two systems generated a heterostructure composite and can be used for any potential application and promotes the making of new materials of high demand. This hybrid structure showed excellent synergism and active interaction between CeO_2_ and NGO which enhanced the physicochemical properties for high HER activity. This coupling of hybrid nanocomposite materials can be further used for any potential energy and environmental-related applications. The results of the present research works were compared with those of the recent research work and are summarized in detail in Table 1. This table shows the results of the present research work comparing the study of CeO_2_ nanoparticles and CeO_2_-based composites for ORR/OER activity.

**TABLE 1:** Comparison of the electrocatalytic activity of 1-CeO_2_@NGO composites with different metal oxides of OER/ORR electrocatalysts that have been recently reported.

Catalyst	Electrolyte	ORR, E_Onset_ (V vs. RHE)	OER, E_Onset_ (V vs. RHE))	Reference
1-CeO_2_@NGO	0.5 KOH	0.925	1.2	This work
CeO_2_/rGO	0.5 KOH	0.915	1.315	Sun et al. (2017)
Co_3_O_4_/NRGO	1 M KOH	0.78	1.55	Kumar et al. (2016a)
FCMO–carbon black	1 M KOH	0.94	1.54	Rai et al. (2020)
CeO_2_–fGO	0.1 M KOH	0.90	1.5	Grewal et al. (2020)
MnVO_*x*_@N-rGO composites	0.1 M KOH	0.98	1.65	Xing et al. (2018)

## 2 Experimental Organization

### 2.1 Materials

Graphite (Merck), sodium hydroxide, sulfuric acid, hydrogen peroxide, hydrochloric acid, cerium (III) chloride hexahydrate, ruthenium oxide, platinum carbide (Pt/C), and Nafion were acquired from Sigma-Aldrich.

### 2.2 Synthesis of Nitrogen-Doped GO (NGO) Sheets

Graphene oxide was synthesized using the improved Hummers method (Kashinath et al., 2017). In brief, sulfuric acid and phosphoric acid (9:1) were added to the mixture of sodium nitrate (1 g) and graphite (2 g). Then, potassium permanganate (6 g) was slowly added gradually and stirred for 2 h. Diluted hydrogen peroxide (10 ml in 100 ml of water) was added and stirred for 2 h. The product obtained was washed with diluted hydrochloric acid (1:10 ml of water) and filtered. The as-prepared GO solution was mixed with 20 ml of N_2_H_4_.H_2_O solution and allowed to stir for 1 h. The homogenous solution was microwave treated for 5 min at 900 W, and then, the treated solution was transferred to Teflon-lined autoclaves. The autoclaves were kept at 150°C for 24 h, and the product obtained after cooling the autoclaves was washed with water and ethanol and dried in a vacuum oven at 80°C.

### 2.3 Synthesis of Hybrid CeO_2_-NGO Composites

**SCHEME 1:**
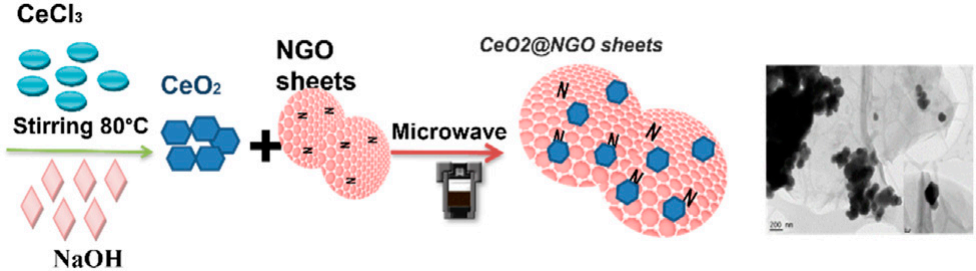
Schematic illustration of hexagonal/isometric ceria NP growth/embedded on the surface of nitrogen-doped graphene oxide sheets.

For the synthesis, 2.46 g of CeCl_3_.7H_2_O in 20 ml of water and 0.4 g of NaOH in water (20 ml) were dissolved and stirred for 30 min. These solutions were gradually added to the NGO solution (as-prepared NGO of 200 mg is dissolved in 200 ml water, sonication for 1 h) and stirred for 2 h at 90°C. This complex solution was treated with microwave irradiation for 5 min at 900 W, and the irradiated solution was transferred to Teflon-lined autoclaves. The autoclaves were kept at 150°C for 24 h, and the product obtained after cooling the autoclaves was washed with water and ethanol, and dried in a vacuum oven at 80°C, named 1-CeO_2_@GO, and two other composites were synthesized as per the above-discussed procedure by varying the concentrations of CeCl_3_ as 0.5-CeO_2_@NGO (1.23 g) and 0.25-CeO_2_@NGO (0.61 g), respectively. For comparison, CeO_2_ was synthesized in the same procedure in the absence of an NGO solution. The schematic illustration of the experimental procedure and the obtained morphology of ceria incorporation on the NGO sheets are shown in Scheme 1.

### 2.4 Physical Characterizations

Rigaku SmartLab–II, (*λ* = 1.544 Å) powder X-ray diffractometer (XRD), was used for studying the crystal phase of synthesized samples. The Raman spectroscopy technique was employed using a Sp action Nicolet NXR FT-Raman spectrometer at 541 nm excitation. Fourier transform infrared spectroscopy was employed using a JASCO FTIR-460 plus spectrophotometer, Japan, to detect the surface functionality. The binding energy of the composites was investigated by X-ray photoelectron spectroscopy (ULVAC-PHI, Japan). The morphological study was conducted by field emission electron microscopy (MODEL-S-3400N, Carl Zeiss, Japan) and high-resolution transmission electron microscopy (Joel/JEM 2100, Hitachi, Japan).

### 2.5 Electrochemical Studies

All electrochemical studies were investigated using a Pine Autolab in the three-electrode system of which glassy carbon (working electrode), Ag/AgCl (reference), and graphite rod (counter electrode) operated in 1 M KOH solution. Cyclic voltammetry (CV) was performed at a sweep rate of 5 mV/s (0.2 to −1.2 V Ag/AgCl) to analyze the physical behavior of the materials in saturated conditions of oxygen and nitrogen bubbling for 30 min. Linear sweep potential voltammetry (LSV) was examined in 0.2 to−1.2 Vs. Ag/AgCl, 5 mV/s. The potentials against Ag/AgCl were converted to the reversible hydrogen electrode (RHE) for calibrations, and the same potential (RHE) was utilized for plotting the graphs of the results.

$${\text{E}}_{\text{ERHE}}={\text{E}}_{\text{Ag/AgCl}}+\text{0}\text{.}997+\text{0}\text{.}059^{\text{∗}}\text{pH}\text{.}$$

#### 2.5.1 Electrode Preparation

The working electrode ink was made from the synthesized catalyst (6 mg) mixed with a 3:1 µL mixture of water and ethanol, followed by sonication for 1 h. Next, 20 µL of Nafion was added and sonicated for 2 h to obtain a homogeneous slurry. Next, 4 µL of the slurry was dropped cast on the glassy carbon electrode and kept for drying at room temperature. The commercially available Pt/C and RuO_2_ electrodes were prepared in the same procedure and tested for electrochemical activities.

## 3 Results and Discussion

### 3.1 X-Ray Diffraction

**FIGURE 1:**
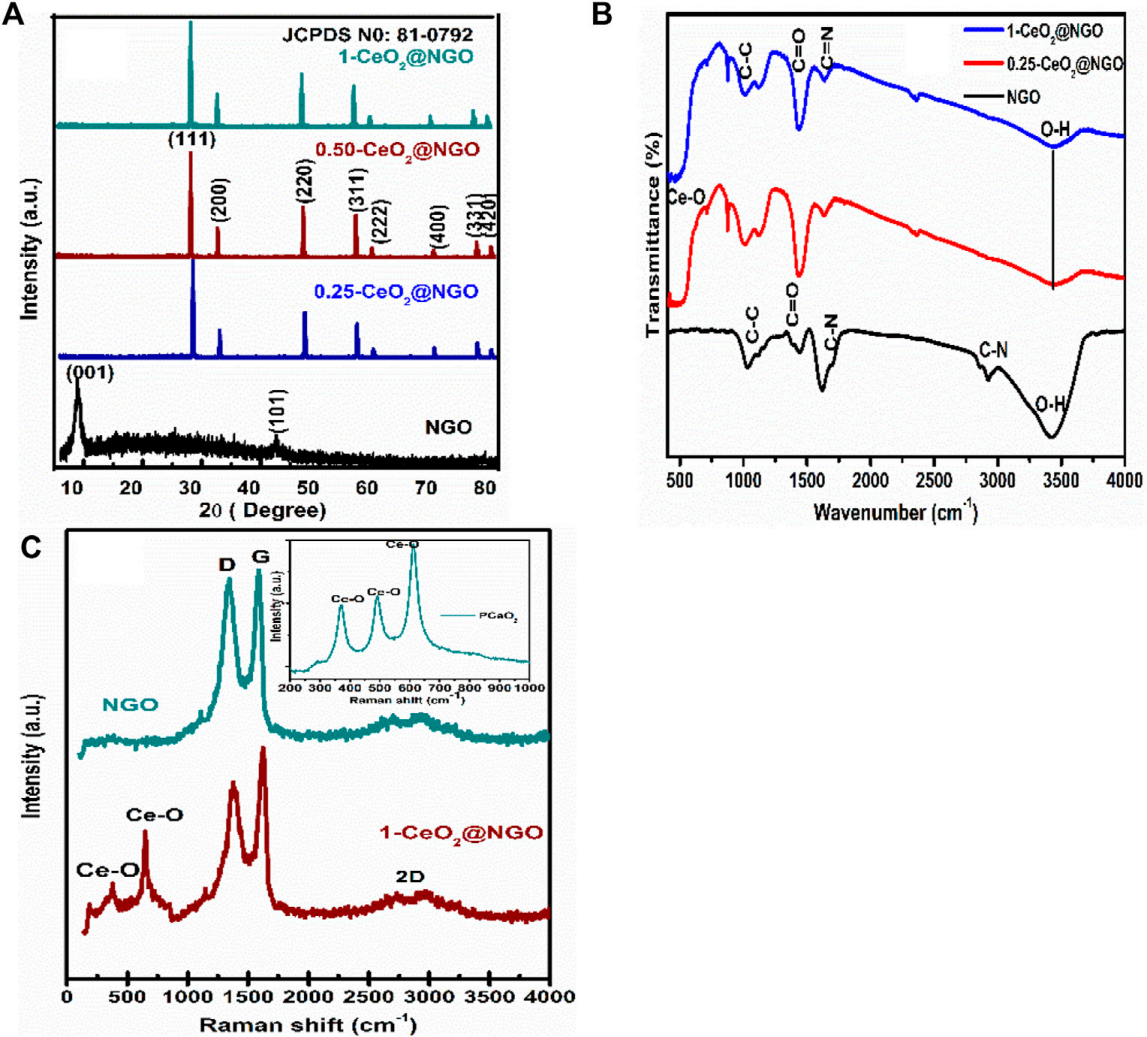
**(A)** XRD patterns of NGO, 0.25-CeO_2_@NGO, 0.5-CeO_2_@NGO, and 1-CeO_2_@NGO. **(B)** FTIR spectra of NGO, 0.25-CeO_2_@NGO, and 1-CeO_2_@NGO. **(C)** Raman spectra for NGO, 1-CeO_2_@NGO, and inset image of pure CeO_2_.

Figure 1A demonstrates the crystal phases of NGO, CeO_2_, and CeO_2_-NGO hybrid composite materials using the X-ray diffraction technique. The oxidation peak of NGO is located at 11.7°(002) and the satellite peak at 43°(101), as shown in Figure 1A. These oxidation peaks disappeared completely in CeO_2_@NGO composites that confirm the presence of CeO_2_ nanoparticles embedded in the layered surface of nitrogen-doped graphene oxide bonding. The peak patterns of ceria nanoparticles observed at 28.7°(111), 33.1°(200), 47.4°(220), 56.4°(311), 58.9°(222), 69.5°(400), and 76.7°(331), respectively, resembled standard diffraction patterns of CeO_2_ (JCPDS 81–0792) exhibiting face-cantered cubic (fcc) as the basic unit for hexagonal structure for 1-CeO_2_@NGO, 0.5-CeO_2_@NGO and 0.25-CeO_2_@NGO composites shown in Figure 1A (Kashinath et al., 2019). The hexagonal formation of cerium oxide nanoparticles can be observed from the results of HR-TEM. Even the piled oxidized structural and diffraction patterns confirm graphene oxide is having less functional groups of hydroxyl and epoxy (Zhan et al., 2014). The *d-spacing* values obtained from diffraction patterns of composite materials are higher than the *d-spacing* of GO, which confirmed the higher reduction of GO and incorporation of the Ce and O bonding interface that enlarges inter-planar spacing. From the diffraction pattern, it can be observed that composites have a sharp intensity which confirmed the highly crystalline formation of CeO_2_ nanoparticles on layered NGO surface, and even the intensity increases as the Ce in CeO_2_-NGO composite increases, which explains the higher amount of cerium oxide nanoparticle formation and having excellent coordination with Ce-O and Ce-O-C. This concluded that the ceria particles have well-organized coordination on the layered surface of nitrogen-doped graphene oxide. Additionally, there is a large increase in the density states and active surface sites in the composites which are confirmed by the functional bonding of FTIR spectra. It also revealed that the iron particle is deeply embedded within, and out of layered graphene sheets can be further confirmed by HRTEM images which support an increase in density states of composites.

### 3.2 FTIR Analysis

Figure 1B shows the functional surface bonding of absorption band deposition of CeO2_2_ NP and CeO_2_-NGO surfaces by the existence of sharp intensive bands at 615 cm^−1^, 540, and 440 cm^−1^ which are due to Ce-O metallic oxide bonds in Figure 1B for 1-CeO_2_@NGO and 0.25-CeO_2_@NGO. The wide bands at 3410 and 1,377 cm^−1^ (O-H stretching), 1732, and 1,080 cm^−1^ are allocated to the C-O and C-OH carboxyl groups (stretching vibrations), respectively, for NGO. The band at 1,615 cm^−1^ confirms the (C=O/C-C) bending of carboxylic vibrations (Peng et al., 2016). The existence of carbon bonding with the nitrogen content absorbed at 1,595 cm^−1^ (C-N), 2,450, and 2,844 (C=N) is observed, and the intensity gradually decreases due to the incorporation of higher participation of ceria particles. The peak intensities of CeO_2_-NGO composite decrease largely which confirmed the higher reduction of GO to form graphene/rGO and in turn maturated layered formation of graphene. The functional groups with carbon and nitrogen bonding confirmed the interfacial bonding of ceria with NGO, and the peak intensity of the OH group of NGO decreases due to the bonding of ceria particles with oxygen atoms and reduction of OH, which leads to the formation of the reduced nature of graphene oxide can be understood by FTIR spectra (Fan et al., 2013; Kumar S. et al., 2016).

### 3.3 Raman Spectroscopy

Raman spectroscopy is an effective spectroscopic technique used to find the phase purity, carbon bonding, and hybridization nature of hybrid complex materials and pure material. Furthermore, it can give evidence about the structural defects of dopants in the synthesized materials. Raman for NGO is situated at 1,355 cm^−1^ (D band) and 1,598 cm^−1^ (G band), respectively, as shown in Figure 1C in which D bands occur due to disorder graphene and G band ordered E_2g_ stretching. The bands at 600 cm^−1^ and 467 cm^−1^ are related to the oxygen vacancies in the CeO_2_ lattice and defects caused by small size effects as shown in Figure 1C, respectively, for the CeO_2_-NGO composite. From the Raman spectra, the combination of ceria nanoparticles on the NGO is clear evidence of the complex heterostructure’s defect-free formation on the sp^2^ hybridized bonding. There is a slight decrease in FWHM of the bands that confirmed the higher density of ceria particle incorporation on NGO sheets. It is a good sign for complex structure formation and supports the microwave-assisted hydrothermal process for the synthesis of heterostructure materials.

### 3.4 Microstructural Analysis

**FIGURE 2:**
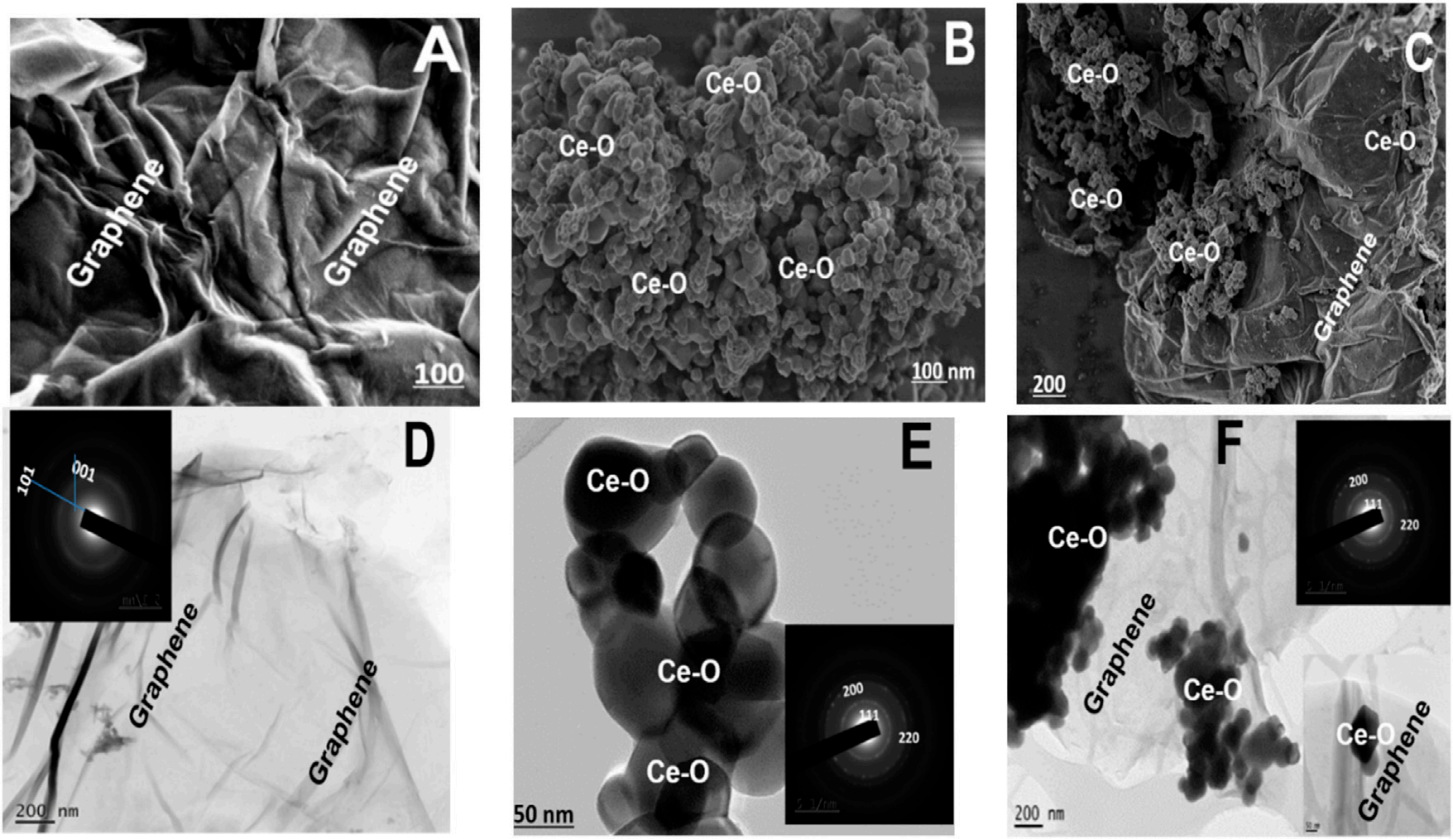
**(A–C)** FESEM images, **(D–F)** TEM, and an inset image of SAED patterns for NGO, CeO_2_, and CeO_2_-NGO.

The synthesized samples were comprehensively investigated and characterized using field emission scanning and transmission electron microscopy techniques. The FE-SEM image of NGO revealed crumpled layered graphene oxide (graphene) sheets having many internal folds and having piled up flakes on which conjugation occurs and decorated uniformly by numerous isometric/hexagonal nanoparticles (Figures 2A–C). The folded crumpled flake-like structure nearly has a porous filtering pattern due to the presence of nitrogen content, and the ceria nanoparticles are nanospheres or possess spherical-like morphology in-depth looks cubic/hexagonal (Figures 2A–C). The combination of ceria and NGO sheets from the FESEM images indicated the decorated ceria nanoparticles on the cripple or wrinkled flakes or sheets. The images revealed that the cerium oxide nanoparticle was occupied on both sides of the layered sheets. To investigate the morphology of composite and further understand the inter contacting cross bonding, crystal information was studied by TEM. With the help of TEM images, it can be understood that the GO is a flat, wrinkled, or crushed long matted sheet (crushed paper) (Figure 2D) with microporous nature; due to the presence of nitrogen doping, the sheets are twisted and possess prolonged surface area. The inset image of Figure 2 showed the SAED designs of two concentric rings of (002) and (101) for NGO and d spacing calculated from SAED that well matches with the crystal information of the XRD analysis. The hexagonal or sphere-like ceria nanoparticles embedded in the surface of layered NGO sheets are shown in Figure 2F (Wu et al., 2012). The SAED pattern shows concentric rings with bright spots, indicating the nanocrystalline (CeO_2_) nature showed inset image of Figure 2E. The lattice fringes of the CeO_2_ crystal showed the d spacing of 0.312 nm which is in good agreement with the d-spacing planes of (111) CeO_2_ from the XRD patterns. From the images, it can be observed that NGO shows a large surface area on which uncountable ceria particles without any aggregation have settled homogeneously that showed a perfect growth and morphology of anchored ceria particles. A clear observation of SAED and d spacing values shows that there is a slight increase in the fringes and spacing which confirmed the occupation of ceria nanoparticles on the layered patterns of NGO. This infers contacting of higher density atoms leads to a higher reduction of graphene oxide and ceria settlement in the deep-layered NGO (Begum et al., 2017).

### 3.5 XPS Analysis

**FIGURE 3:**
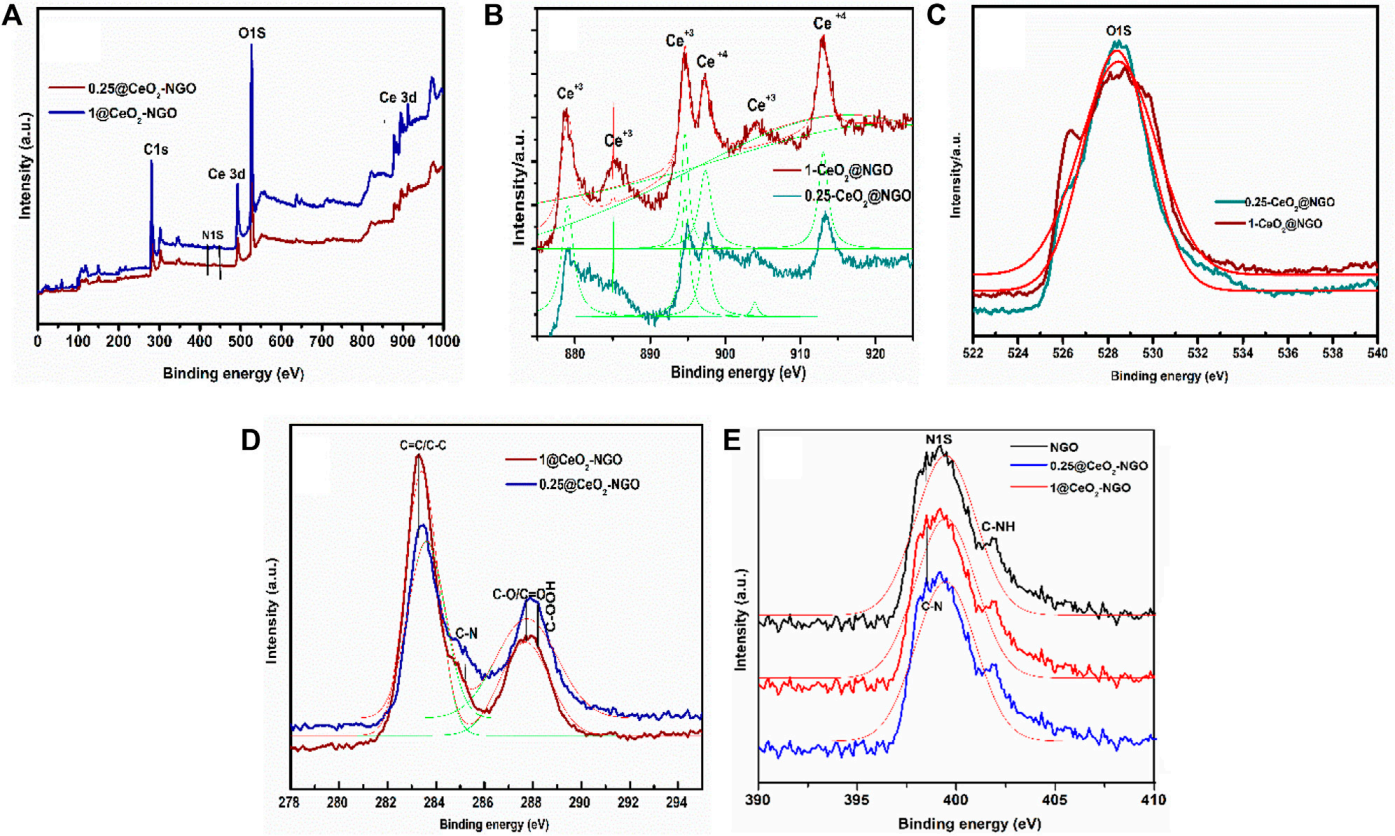
**(A)** XPS survey scan of 0.25-CeO_2_@NGO and 1-CeO_2_@NGO. **(B–E)** High-resolution detailed scan of Ce 3d, O1S, C1S, and N 1S, respectively, for 0.25-CeO_2_@NGO and 1-CeO_2_@NGO

The chemical states and element-binding energies in the prepared composite were detected by X-ray photoelectron spectroscopy (XPS) shown in Figure 3. The full-fledged survey spectrum consisting of Ce, O, N, and C is identified in Figure 3A for 0.25-CeO_2_@NGO and 1-CeO_2_@NGO, respectively. The high-resolution deconvoluted C1S spectrum contributes to carbon bonding of 285.5 eV (C-N/C-O), 284.6 eV (C=C/C-C), and 284.9 eV (C=N) which confirmed the perfect blend of nitrogen-doped graphene oxide association of C-N can actively promote the better performance as donor-acceptor active sites and the carbon matrix with Ce clusters; the C-O association enhances the catalytic activity (Dowding et al., 2012; Murugan et al., 2018). These oxidation peaks and bonding suggested the existence of oxygen functionality on the surface of GO sheets that supported the conduction and electron transfer very rapidly and with a higher amount of current. The carbon atoms were linked with nitrogen atoms in the composite materials which enhanced the performance of electrocatalytic activity due to excess lone pair of an electron from nitrogen which supports as donor-acceptor for excellent charge mobility. The existence of nitrogen content in composite, the high-resolution spectra show pyrrolic N at 400.1 eV, and graphitic peaks at 401.2 eV of nitrogen confirmed the incorporation of nitrogen in the matrix of graphene in the processing of composite; the nitrogen content intensity is decreased due to overlapping of Ce nanoparticles on the surface of NGO, which in turn reduced the bonding of n-graphene, and Ce-C-O is increased. There are small differences in the binding energy of nitrogen that forms bonding with Ce attracting electron transfer from Ce that results in lower energy and turn conjugation with graphene oxide. As the concentration of ceria increases in the composite, the decrease in nitrogen spectra confirmed the higher number of vacant places occupied by ceria nanoparticles. The high-resolution oxidation spectra of cerium binding energy splitting are located at 885.4 eV (Ce 3d) and 904.2 eV (Ce3d) final states of Ce3d (3d_5/2_ and 3d_3/2_). The two peaks at 916.1 eV (associated with the Ce 3d_3/2_) exhibit the presence of tetravalent Ce (IV) and 900.4 eV is from Ce 3d3/2 ionization, and 882.0 eV from Ce 3d5/2 ionization for Ce^3+^ and Ce^4+^. In addition, the peaks located at 906.5 and 887.9 eV are associated with the Ce3d94f0O2p6 a “shakedown” states the O2p level to the Ce 4f level in the excited state.

### 3.6 Electrochemical Studies

**FIGURE 4:**
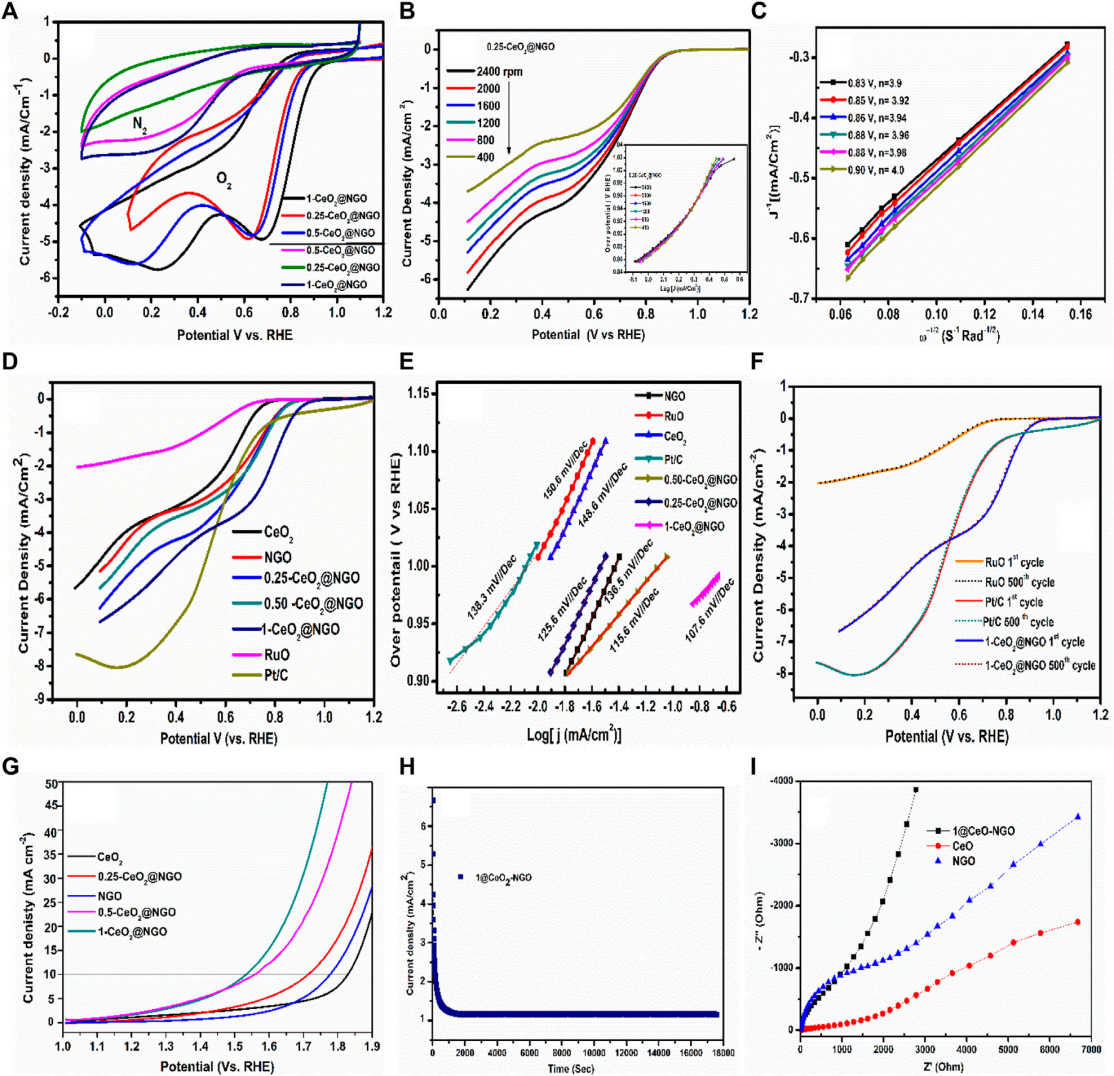
**(A)** CV in oxygen and nitrogen-saturated conditions, CV in 0.5 M KOH, scanning rate 100 mVS^−1^ for NGO, 0.25@CeO_2_-NGO, 1@CeO_2_-NGO, and RuO_2_. **(B)** LSV graph of 0.25@CeO2-NGO with different rpm values at 5 mVS^−1^ (inset image Tafel slope for 0.25@CeO_2-_NGO). **(C)** K-L plot for 0.25@CeO2-NGO. **(D)** Polarization curves of pure CeO_2_, NGO,0.25@CeO_2_-NGO, 0.50@CeO_2_-NGO, 1@CeO_2_-NGO, RuO_2_, and Pt/C at 2000 rpm. E) Tafel slope for CeO2, NGO, 0.25@CeO_2_-NGO, 0.50@CeO2-NGO, 1@CeO2-NGO, RuO_2_, and Pt/C at 1,600 rpm. **(F)** Stability test for RuO_2_, Pt/C, and 1-CeO_2_@NGO at 2000 rpm, and **(G)** OER studies of pure CeO_2_, NGO,0.25@CeO_2_-NGO, 0.50@CeO_2_-NGO, and 1@CeO_2_-NGO. **(H)** Chronoamperometric study for 1@CeO_2_-NGO at 0.9 V. **(I)** EIS spectra of pure CeO2, NGO, and 1-CeO_2_@NGO.

The electrochemical activity of the as-prepared catalyst materials was first evaluated using the cyclic voltammetry technique in both oxygen and nitrogen saturated conditions operated at 100 mVs^−1^ in 0.5 M KOH electrolyte. From the CV studies, it can be predicted that rectangular patterns with high redox nature peaks illustrated the anodic potential tendency which is nearly having similar trends in oxygen-saturated conditions. From a close observation, the redox nature of ceria composites is increasing slightly as the molar concentration of ceria in composite increases. This indicates the higher redox states and in turn, shows generating a high current with active potential sites for oxygen evolution or reduction reaction which can be seen in the nitrogen atmosphere (Figure 4A). With respect to nitrogen-saturated conditions, the catalyst materials show a fine and broad redox peak in oxygen conditions, and the amount of current production is higher, and 2 times greater than that is observed for NGO, 0.25@CeO_2_-NGO, and 1@CeO_2_-NGO (Figure 4A). Also, the overpotential of the catalyst materials is highly cathodic which is prominently significant for oxygen reduction and evolution reaction. CV for ruthenium oxide (RuO_2_) was analyzed in the same conditions for comparison of the catalyst performance, as can be seen in Supplementary Figure S1. Electrochemical surface area analysis was calculated to determine the efficiency of catalyst with respect to loading on the electrode. The surface areas of the pure CeO, NGO, and 1@CeO-NGO were estimated from the graph plot between the I Vs V^1/2^ using Randle’s-Sevcik eq

$$\text{I}=\left(2\text{.}69\text{×}105\right){\text{ACD}}^{1/2}{\text{n}}^{3/2}v^{1/2}$$

where A (cm^2^) = surface area of the electrode, C = concentration of 0.5 M KOH solution, D = diffusion coefficient of the analyte (KOH), n = number of electrons per molecule or ion or number of Faradays per mol of molecules or ions taking part in the oxidation or reduction reaction, and ν = scan rate. The surface areas of pure CeO, NGO, and 1@CeO-NGO, respectively, are 0.0607, 0.0976, and 0.1161 cm^2^. An insight study of the electrochemical performance of the catalyst was further examined using the linear sweep voltammetry polarization technique to understand the electron transferability tendency with a scanning rate of 5 mV/S in the potential range of 0.2 to−1.2 V vs. RHE. The onset overpotential and current density produced by varying the speed of rotation from 400 to 2,400 rpm in oxygen-statured electrolyte conditions are investigated (Figure 4B). An onset potential of 0.9 V (RHE) with a current generation of 3.75 mA at 400 rpm was observed from LSV. In addition, as the speed of rotation is increased, a stable onset potential with an increasing trend in the current generation is observed at 2,400 rpm 6.75 mA, which is very high compared to pure CeO_2_, NGO, and other previous reports. The LSV of 0.50@CeO_2_-NGO and 1@CeO_2_-NGO with different rotation speeds and the respective Tafel slope exhibiting the linearity is shown in Supplementary Figure S2. Another important aspect of ORR activity is electron transferability and its efficiency for long-term ability. Also, the numbers of the electron pathway (4 electron transfer) are calculated using the K-L method. The multiple electron charge transfer reaction has a direct four-step pathway involving four-electron path transfer to generate a higher current density. The oxygen is reduced in a singular four-step electron mobility charge transfer pathway which play an important role in oxygen reduction than the two-electron pathway transfer. The Koutechy–Levich (K-L) method is adopted to know the catalyst electronic charge transfer tendency from the following equation as $\frac{1}{J\;}=\frac{1}{Jkin}+\frac{1}{\;B\sqrt{\omega }}$ , where B is constant (J_K_) = 0.62 n. F. D_o_^2/3^. ν^−1/6^. C, in which J is the current density, and J_k_ is the kinetic current density, ω is rotation kinetic speed, n is the number of electrons transferred number, F is Faraday constant (F = 96,485 C mol^−1^), oxygen diffusion coefficient (D = 1.9 × 10^−5^ cm^2^ s^−1^), ν is the kinematic viscosity (ν = 0.01 cm^2^ s^−1^), and C_o_ is the concentration of oxygen of solution (1.2 × 10^−6^ mol cm^−3^). A plot against J^−1^ and ω^−1^ with the respective potential and the slope of the graphs gives a four-electron pathway. The K-L plots give the first-order kinetics and electron transfer at the different potentials for ORR can be calculated shown in the inset image of Figure 4C. From Figure 4C, the values of electron transfer numbers are 3.6, 3.8, 3.85, 3.95, 3.98, and 4, respectively, for 0.25@CeO_2_-NGO to the recorded potential (Lefèvre and Dodelet, 2003; Yang et al., 2015). The calculated values of electron processing are very near to 4, which concludes the tendency of four electron transfer processes by synthesized materials which are having a stable potential tendency to produce excellent current, faster kinetic, and rapid transfer of electron ability for energy application (Cracknell et al., 2008; Gewirth and Thorum, 2010). The Tafel slope gives the insight efficiency and tendency of materials toward the electrochemical performance. The linear intercept of the slope obtained from the Tafel slope revealed that the conduction of electron transferability is directly proportional to potential and follows the four pathways of electron number transfer. The Tafel slope of 110 mV/Dec is observed in the potential range of 0.8–1 V (inset image of Figure 4B), and a decreasing trend in the value of Tafel slope is observed for a higher amount of ceria in the composite, as can be seen in Supplementary Figure S1. To understand the effect and influence of ceria nanoparticles in the composite, the LSV studies were evaluated for different hybrid composites synthesized by varying the concentrations of cerium oxide in the same conditions, and for comparison of commercially available RuO_2_ and Pt/C were also tested. Figure 4D shows the polarization graph of CeO_2_, NGO 0.25@CeO_2_-NGO, 1@CeO_2_-NGO, RuO_2_,_,_ and Pt/C at a scanning rate of 5 mVS^−1^. From the LSV graph, the overpotentials are observed at 0.75, 0.825, 0.85, 0.9, 0.925, 0.65, and 0.815 V, respectively, for CeO_2_, NGO, 0.25@CeO_2_-NGO, 1@CeO_2_-NGO, RuO_2_, and Pt/C, respectively. The Tafel slope values plotted between the log of current density and potential voltage shown in Figure 4E confirmed the insight tendency of the composite. A deep look into the polarization curve at the generated current density between 0 and −1 gives insight tendency of individual material characteristics of electron transfer and conduction. It is observed at the potentials of 0.75 V (150.6 mV/dec), 0.65 V (148.6 mV/dec), 0.825 V (138.3 mV/dec), 0.815 V (136.5 mV/dec), 0.85 V (126.5 mV/dec), 0.9 V (115 mV/dec), and 0.925 V (107 mV/dec) for RuO_2_, CeO_2_, NGO, Pt/C, 0.25-CeO_2_@NGO, 0.50-CeO_2_@NGO, and 1-CeO_2_@NGO, respectively, and similarly the current generated by ceria doped NGO is higher and higher than Pt/C, RuO2, NGO, and CeO_2_ that signify the importance of coupling of the ceria nanoparticle with NGO, which exhibits an excellent synergic effect. The onset potential and current generated from the hybrid nanocomposite synthesized using the microwave and hydrothermal coordination system for ORR activity is best and better than the reported values of Lihua Sun for CeO_2_-rGO with 138.6 mV/dec, and Weidong Peng reported an onset potential of 0.75 V (RHE) with 140 mV for CeO_2_-rGO. The LSV studies clearly showed the combination of cerium oxide nanoparticles on the surface of nitrogen-doped graphene oxide and demonstrated excellent electrochemical activity with a lower onset potential and generating a higher amount of current. The power generation can be increased further by varying the higher amount of ceria and functionality of GO. With a potential of 0.2, the current produced by Pt/C started to decrease and led to a gradual fall in current and voltage, but the nanocomposite showed increase in current and no change in performance. Thus, it is understood that the composite has excellent stability and performance due to a combination of microwave and hydrothermal processing which is favorable for electrochemical properties. To understand the efficiency and stability of the catalyst, the LSV was performed in 0.5 M KOH for the first cycle and tested up to 500 cycles in potential voltage of 1.2 to −0.2, respectively, for 1-CeO_2_@NGO, RuO_2_, and Pt/C shown in Figure 4F for ORR activity. The cyclic performance studies revealed that the catalyst shows no difference in the onset potential, and the current density generated remains constant throughout from 1st cycle to 500 cycles without losing its stability and efficiency and exhibits excellent activities. This coupling technique of synthesized materials can be used for long cycles and without losing its intrinsic electrochemical performance and is highly motivated for the replacement of Pt-based materials for large production. Furthermore, ceria nanoparticle incorporation on the surface of GO, rGO, and NGO sheets was investigated for ORR/OER studies at 1,600 rpm. The comparison of ceria loading on different graphene oxide structures was studied in deep, and the investigation revealed that the ceria loading on NGO sheets gives a better ORR/OER activity when compared to ceria loading on GO and RGO, respectively, and are shown in supplementary information Supplementary Figure S1. Interestingly, the synthesized electrocatalyst materials showed excellent OER performance with a lower onset potential and high current as out in 0.5 M KOH solution. In detail, the OER activity of the catalyst materials was performed in the same electrochemical condition with a scan rate of 5 mVs^−1^ for pure CeO_2_, NGO sheets, 0.25@CeO_2_-NGO, 0.5@CeO_2_-NGO, and 1@CeO_2_-NGO, respectively, as shown in Figure 4G (Yang et al., 2016). Concerning the image from Figure 4G, the LSV polarization curves show an onset/over the potential of 1.77, 1.61, 1.52, 1.35, and 1.2 V vs. RHE, respectively, for pure CeO_2_, NGO sheets, 0.25@CeO_2_-NGO, 0.5@CeO_2_-NGO, and 1@CeO_2_-NGO is higher than precious catalyst materials as Pt/C (1.16 V vs. RHE), RuO_2_ (1.01 V vs. RHE) and Ir/C (0.92 V vs. RHE) reported by Shanmugam (Ganesan et al., 2015), (Lefèvre and Dodelet, 2003; Parvez et al., 2012). From the onset potential, it can be concluded that this synthesis approach shows a low overpotential with a higher current density value. The chronoamperometric study was conducted in the same electrolyte conditions for 1@CeO_2_-NGO with an onset potential of 0.9 V as shown in Figure 4H. To understand the charge-transfer behavior and electrical tendency of as-prepared heterostructure nanocomposite, electrodes and electrolytes were investigated using electrochemical impedance spectroscopy studies (EIS) as shown in Figure 4I, respectively, and information regarding the charge/solution resistance, impedance, and constant phase elements were tabulated, as shown in Supplementary Table S1. The diffusion phenomena and interfacial charge transport nature at the electrode/electrolyte were obtained from the Nyquist plot. From the Nyquist plot, the electrocatalysts comprise semi-circular arc in the higher-frequency region and a straight line in the lower-frequency region. The diameter of the semi-circular region represents the charge transfer resistance at the electrode-electrolyte interface, and the straight line displays the diffusion characteristics of the electrolyte at the electrode surface. The EIS spectra were fitted with an equivalent circuit consisting of solution resistance (Rs), charge-transfer resistance (Rct), Warburg impedance (Zw), and constant phase element (CPE). The semicircle intercepting the real axis is a combination of both charge transfer resistance Rct (Guan et al., 2018) and ionic resistance of electrolyte Rs (Maheswari and Muralidharan, 2016). Rs consists of bulk electrolyte solution resistance and electron transfer resistance, whereas Rct can be ascribed to change transfer resistance at the electrode-electrolyte boundary (Maheswari and Muralidharan, 2015). From the electrochemical results, it is clearly indicated that the incorporation of ceria NP on the *in situ* nitrogen-doped graphene oxide sheets increased the ORR/OER activity scientifically. The NGO sheets facilitate more active sites and reduce the activation energy which in turn boosts the charge transfer kinetic of OER/ORR.

The incorporation of cerium oxide nanoparticles on the layered surface of NGO onset potential slowly increases and right shifts to more anodic potential with higher power generation. The current generated was found to be increased as the content of ceria in composite increased which steadily confirmed the direct relation of ceria and NGO and shows the better synergic affect performance. The increase of onset potential toward higher anode due to nitrogenous graphene oxide coupling with cerium oxide-controlled morphology with ultrafine particles is observed. The interfacial tendency of NGO and ceria with increases in atoms concentration increases the rapid charge transfer at the interfaces (Kalubarme et al., 2012). The reduction of graphene oxide and interfacing between the nitrogen and reduced form of graphene oxide retains a minor quantity of oxygen and functional containing groups at edges, and the remaining groups have a higher intrinsic activity than those terminating the flat (001) surface. This tendency of NGO promotes the making of active oxidative sites for interaction of ceria nanoparticles, which enables the entire surface with an electron-rich surface that generates and transfers the conduction rapidly (Frank et al., 2009, 2011). The combination of microwave and the hydrothermal process will enhance a stronger electronegativity of nitrogen and its conjugation between the carbon and nitrogen as well as the ceria particles, the additional lone pair of nitrogen enhanced the performance. This coupling of heterostructures materials using two different technique facilities in higher power production and developing more and more interactive sites on the surface of NGO is observed, which acts tentacles with high electro conduction behavior. The microwave irradiation makes the components defect-free and removes the week van der Waals force or week bonded forces on the surface and in the composite. The hydrothermal process helps in the re-crystallization of sites in well-ordered patterns which develops more interaction and bonding between the functional groups of NGO and cerium oxide nanoparticles further developing the strong and active site of Ce-N-O and Ce-N-C, with additional epoxy which stimulates the density of the material. Thus, the coupling of heterostructure materials with microwave and hydrothermal synthesis process plays a vital role and enhancement in the physicochemical characteristics that enrich the electrocatalyst activities (Wu et al., 2018; Kashinath, 2021). The active interaction of graphene oxide functional groups with nitrogen provides a surplus electron density, and pi-pi interaction results in strong coupling in the composite sustainable for any environmental conditions. The ceria particles settled on the nitrogenous graphene sheets supports anchoring for rapid transferability. The nitrogenous carbon content of graphitic and pyrrolic N bonding is effectively active for the oxygen reduction reaction. A higher amount of Ce (III) than the Ce (IV) and the coexistence of oxidized nanostructure without any zero-valent ceria particles confirmed from XPS spectra which limits the defects and the coordination of ceria nanoparticles coordination with highly graphitic N content shows an energetic role in the ORR performance. The graphitic N and pyridine N atom’s coordination with Ce increases the catalytic activity compared to pure CeO_2_ and NGO. This coupling and coordination between Ce and NGO enhanced the activity of electrochemical performance and showed a good synergism (Liu et al., 2010; Kim et al., 2011). The increase in interlayer spacing with a high amount of nitrogen doping and further decoration of Ce nanoparticle increases the distance between the spacing, thus enhancing high surface and diffusion kinetics on the surface. The nitrogen content helps facilitate higher electron transfer ability and higher current generation and the excellent tendency of acceptor-donor promotion to a higher level. This characteristic provides a rapid and higher tendency of conductivity which added an advantage to producing non-precious bifunctional oxygen reactions and for other energy-related applications. It is believed that this coupling of techniques provides more active nanocrystal sites of Ce-N-C and Ce-N-O and functionally attached to the exfoliated large surface of NGO sheet in generating higher current, power, and elevation density of materials, the surface to volume ratio, and exhibits excellent synergistic effect.

## Conclusion

In conclusion, we have successfully synthesized a highly crystalline hexagonal ceria nanoparticle decorated on an *in situ* nitrogen-doped graphene oxide sheet *via* a microwave-assisted hydrothermal method. The heterostructural nanocomposites were successfully tested for bifunctional OER/ORR activity in KOH solution. CeO_2_@NGO exhibited superior physicochemical properties and exhibited excellent synergetic effect. The dominant phase with a smaller particle size of CeO_2_ was embedded on the encrusted surface of NGO and was confirmed by microscopic techniques of FESEM and HR-TEM. Heavily nitrogen-doped GO ornamented with ceria nanoparticles is an effective and efficient way of acting as a higher electron channel which enables the higher OER/ORR activity. The heterojunction composite showed remarked bifunctional activity (OER/ORR) performance better than pure CeO_2_ and NGO. The enhancement of electrochemical and electron diffusion activity originated from the excellent synergism. The coordination and interlinkage of CeO_2_ and NGO enhanced the physicochemical properties, making the nanocomposites robust for multifunctional materials for energy and environmental concerns. This work opens a novel approach to fabricating an efficient electrocatalyst for overall water splitting and other electrochemical studies.

## Data Availability

The original contributions presented in the study are included in the article/Supplementary Materials; further inquiries can be directed to the corresponding author.
