# Supplementary material for: Ceria Boosting on In Situ Nitrogen-Doped Graphene Oxide for Efficient Bifunctional ORR/OER Activity
Source: Front Chem. 2022 Jun 24;10:889579. doi: 10.3389/fchem.2022.889579 (PMC9263449; doi:10.3389/fchem.2022.889579)
Supplement: Supplementary file 1 [file DataSheet1.docx]

**SUPPLEMENTARY INFORMATION**

# **Ceria boosting on in-situ nitrogen-doped graphene oxide for efficient bifunctional ORR/OER activity**

**Electrochemical studies**





**Figure S1**. Cyclic voltammetry of RuO^2^ in oxygen and nitrogen saturated conditions at 5 mVS^-1^ in 0.5 M KOH.












**Figure S2** (C-D) Polarization curves and (B-E) Tafel slope at 5 mVS^-1^ at different speed rotation from 400 to 2400 rpm for [0.5@CeO-NGO](mailto:0.5@CeO-NGO) and1@CeO-NGO in 0.1 M KOH







**Figure S3** A) ORR and B) OER Polarization curves of 1-CeO_2_@NGO, 1-CeO_2_@rGO, 1-CeO_2_@GO at 16000 rpm
